# Decrease expression of microRNA-20a promotes cancer cell proliferation and predicts poor survival of hepatocellular carcinoma

**DOI:** 10.1186/1756-9966-32-21

**Published:** 2013-04-18

**Authors:** Ming-Qi Fan, Chi-Bing Huang, Yan Gu, Ya Xiao, Jin-Xin Sheng, Lin Zhong

**Affiliations:** 1 Transplantation Center, Xinqiao Hospital, The Third Military Medical University, Chongqing 400037, China; 2 Department of General Surgery, Shanghai First People’s Hospital, School of Medicine, Shanghai Jiao Tong University, Shanghai 200080, China

**Keywords:** MicroRNA-20a, Hepatocellular Carcinoma, Recurrence, Prognosis, Liver transplantation

## Abstract

**Background:**

Growing evidences indicate microRNAs play important roles in cancer development, progression, metastasis and may constitute robust biomarkers for cancer prognosis. The aim of this study was to identify the clinical and functional association of microRNA-20a (miR-20a) in hepatocellular carcinoma (HCC).

**Methods:**

MiR-20a was detected using Taqman real-time polymerase chain reaction. Kaplan-Meier and Cox proportional regression analyses were utilized to determine the association of miR-20a with survival of patients. The potential functions of miR-20a on proliferation were evaluated by proliferation and flow cytometry analysis. The direct target gene of miR-20a was also identified by luciferase reporter assays.

**Results:**

MiR-20a was lower in primary HCC than normal liver, and were further decreased in those with post-liver transplantation (LT) HCC recurrence compared with those with non-recurrence (p = 0.001). Patients with lower miR-20a expression had significantly poorer recurrence-free survival (RFS, Log rank p < 0.001) and overall survival (OS, Log rank p < 0.001). Multivariate analysis revealed that lower miR-20a was an independent predictor of poor prognosis. MiR-20a restoration could suppress HepG2 and SMMC-7721 cells proliferation and induce cell cycle G1 arrest and apoptosis. Subsequent investigations revealed that miR-20a directly targeted myeloid cell leukemia sequence 1 (Mcl-1) and reduced the endogenous protein level of Mcl-1 in HCC cells.

**Conclusions:**

MiR-20a is decreased in HCCs and correlates with HCC recurrence and prognosis. Down-regulation of miR-20a increases the proliferation abilities of HCC cells. Our findings suggest miR-20a may represent a novel potential therapeutic target and biomarker for survival of HCC patients.

## Introduction

Hepatocellular carcinoma (HCC) is the third leading cause of cancer-related deaths in the world, with an estimated 21 000 new cases diagnosed and accounting for ~700 000 deaths annually [1]. To date, surgery remains the best prognostic tool for long-term survival of HCC patients; however, more than 80% of patients with HCC have underlying cirrhosis, and of these patients, only 10% to 15% are potentially resectable [2]. The rest are unresectable because of size, location, or severity of underlying liver disease. Liver transplantation (LT) probably offers a therapeutic option for these HCC patients, especially in cirrhotic patients without local or distant metastasis of HCC [3].

However, the risk of HCC recurrence is remain the major concern in patients transplanted for HCC. Understanding the molecular alteration that distinguish progressive from nonprogressive HCC will allow the identification of novel prognositic markers or therapeutic targets and may be useful to guide the post-LT surveillance. Recently, increasing evidences indicate that microRNAs can be potential tools for cancer diagnosis and prognosis [4]. MicroRNAs are small noncoding RNA gene products about 22 nt long that are found in divers organisms and play key roles in post-transcriptional regulation of targeted gene expression through sequence-specific interaction with the 3^′^-untranslated region (3^′^-UTR) of targeted genes [5]. MicroRNAs are important players in basic cellular functions such as, embryonic development, cell growth, apoptosis, and differentiation. However, dysregulation of microRNA is also common in various cancers. The dysregulated miRNAs play roles in carcinogenesis or tumor progression by altering the normal gene expression patterns.

MicroRNA-20a (miR-20a) was found to be down-regulated in several solid tumors, such as breast cancer [6] and pancreatic carcinoma [7], while miR-20a were found to be significantly up-regulated in colon adenocarcinoma [8] and gliomas [9]. This indicates that miR-20a may be a tissue specific microRNA. On the other hand, miR-20a has been shown to inhibit proliferation and metastasis of pancreatic carcinoma cell by directly down-regulating Stat3, that is activated in primary pancreatic cancer and is involved in various physiologic functions, including apoptosis, cell cycle regulation, angiogenesis, and metastasis [7]. Bioinformatic target gene predictions followed by experimental target gene validations revealed that miR-20a act in a common manner by down-regulating an overlapping set of target genes, including E2F family, cyclin-dependent kinase inhibitor CDKN1a/p21, which were mostly involved in regulation and execution of G1/S transition in the cell cycle [10]. Our previous study has shown that miR-20a was correlated with HCC recurrence [11]. However, the biological functions of miR-20a in HCC were not clear and the association between miR-20a and HCC prognosis following LT has not been evaluated yet.

In our current study, we evaluated miR-20a expression levels in 100 formalin-fixed paraffin-embedded (FFPE) tumor tissues of patients with HCC and found that miR-20a was significantly down-regulated in HCC. Based on gain-of-function approach, we proved that miR-20a could inhibit HCC cell proliferation and induce apoptosis in vitro. Furthermore, the Mcl-1 (myeloid cell leukemia sequence 1) protein, an antiapoptotic member of Bcl-2 family, which is usually overexpressed in a variety of human cancers including HCC [12] and plays a pivotal role in protecting cells from apoptosis and tumor carcinogenesis [13], was identified as a direct target of miR-20a. This result provided a possible regulation pathway for Mcl-1 and a candidate target for HCC treatment. We also demonstrated that miR-20a may serve as a potential therapeutic target and biomarker for survival of HCC patients following LT.

## Materials and methods

### Patients and tissue samples

A total of 100 patients undergoing LT for HCC and the follow-up data about the patients in this study were obtained from Liver Transplantation Surgery, Shanghai First People’s Hospital, Shanghai, China, from 2002 to 2007. All the patients were followed until December 2010. The median recurrence-free period was 12 months for patients with HCC recurrence and 64 months for patients without HCC recurrence. All of these 100 patients fulfilled the Up-To-Seven transplantation criteria for HCC [14] and none of them had macro-vascular invasion. HCC samples were from the FFPE tissue blocks and the normal liver tissues were from the liver hemangioma resection. The clinicopathological features of patients were summarized in Table 1. Pre-LT serum AFP level stratification was according to the previous study [15]. All patients provided informed consent according to the protocols approved by the Institutional Review Boards of Shanghai First People’s Hospital.

**Table 1:** Clinical characteristics of the 100 HCC patients according to high- or low miR-20a expression level

**Parameter**	**N**	**Patients with low miR-20a expression**	**Patients with high miR-20a expression**	***P*-value**
Age	100	57.820 ± 7.330	53.64 ± 8.341	0.212^†^
Sex				
Male	84	44	40	0.585^‡^
Female	16	6	10	
Underlying liver disease				
HBV	95	47	48	1.000^§^
others	5	3	2	
Cirrhosis				
Yes	95	47	48	1.000 ^§^
No	5	3	2	
Tumor stage				
I + II	66	32	34	0.673^‡^
III	34	18	16	
Histologic grade				
Differentiated	88	41	47	0.065^§^
Undifferentiated	12	9	3	
Milan criteria				
In	55	24	31	0.159^‡^
Out	45	26	19	
Tumor size (cm)				
≤5	60	24	36	0.014^‡^
>5	40	26	14	
Multinodular				
Yes	43	25	18	0.034 ^‡^
No	57	25	32	
Micro-vascular invasion				
Yes	22	16	6	0.016 ^‡^
No	78	34	44	
pre-LT serum AFP level				
≤400 (ng/ml)	63	30	33	0.534 ^‡^
>400 (ng/ml)	37	20	17	
Overall survival	42/100	11/50	31/50	-
HCC recurrence	58/100	37/50	21/50	-

NOTE: AFP, alpha-fetoprotein. ^†^Unpaired student *t* test; ^‡^chi-square test; ^§^Fisher’s exact test.

### Cell culture and transfection

All the cell lines used in this study were purchased from the cell bank of the Chinese Academy of Sciences and grown in DMEM (GIBCO, Grand Island, NY), supplemented with 10% fetal bovine serum (Sigma-Aldrich, St Louis, MO), 2 mM glutamine, 100 U of penicillin/ml and 100 μg of streptomycin/ml (Cambrex, Verviers, Belgium). All cells were incubated at 37°C in a humidified chamber supplemented with 5% CO_2_. Control negative oligonucleotide, and double-stranded RNAs that mimic endogenous precursor miR-20a were purchased from Ambion (Ambion, Austin, TX) were transfected into cells using Oligofectamine (Invitrogen, Carlsbad, CA) according to the manufacturer’s instruction.

### RNA isolation and Taqman real-time PCR

Total cellular RNA with efficient recovery of small RNA was isolated from 10 × 15 μm section of FFPE tissues using RecoverAll™ Total Nucleic Acid Isolation Kit (Ambion, Forest City, CA) according to the manufacturer’s instructions. Taqman real-time polymerase chain reaction (PCR)-based detection of mature miR-20a was performed by the TaqMan microRNA assays (Ambion, Forest City, CA) as described previously [16]. U6 small RNA was used as an internal control for normalization and quantification of miR-20a expression. All experiments were done in triplicate.

### Cell proliferation assay

Cell proliferation assay was done using cell Titer 96 Aqueous one Solution Cell Proliferation Assay (Promega, Madison, WI) according to the manufacturer’s protocol.

### Cell cycle analysis

HepG2 and SMMC-7721 HCC cells were transfected as described above. After incubated for 48 h, the cells were typsinized, washed with PBS twice, and then fixed with cold 75% ethanol at 4°C overnight. The fixed cells were centrifuged, resuspended in PBS at 1 × 10^6^ cells/ml and incubated with ribonuclease A and propidium idide (PI) at 37°C for 30 min, then followed by flow cytometric analysis using FL2 histogram of a flow cytometer (FACSort; Becton Dickinson, San Jose, CA).

### Apoptosis analysis

Cells were harvested at the above indicated time points, at least 5 × 10^5^ cells were recovered by centrifugation for evaluation of apoptotic cells with the use of double staining with annexin V–fluoresein isothiocyanate (annexin V–FITC) and propidium iodide (PI) (BioVision, St Pete Beach, FL) according to the manufacturer’s instructions, followed by flow cytometric analysis with the use of the FL-1 and FL-3 channels of a flow cytometer, where apoptotic cells are defined as annexin V + and PI-.

### Luciferase activity assay

For luciferase reporter assay, HEK293T cells were cultured in 48-well plates and then cotransfected with 10 ng of either pGL3cm-MCL-1-3^′^UTR-WT or pGL3cm-MCL-1-3^′^UTR-MUT, 30 pmol of miR-20a precursor or negative control oligonucleotides, and 2 ng of pRL-TK (Promega, Madison, WI). Transfection was done using Oligofectamine (Invitrogen, Carlsbad, CA) according to the manufacturer‘s protocol. Cells were collected 48 h after transfection and analyzed using the Dual-Luciferase Reporter Assay System (Promega, Madison, WI). Experiments were done independently in triplicate.

### Western bolt analysis

Cell lysates were resolved by sodium dodecyl sulfate-polyacrylamide gel electrophoresis, transferred to nitrocellulose membrane (Bio-Rad, Hercules, CA) and blocked in phosphate-buffered saline/Tween-20 containing 5% nonfat milk. The membrane was incubared with antibodies for Mcl-1 (Abcam, Cambridge, MA; 1:1000) or GAPDH (Sigma, St. Louis, MO; 1:5000). The antigen-antibody comples was detected using enhanced chemiluminescence (Pierce, Rockford, IL).

### Immunohistochemical (IHC) staining

Paraffin-embedded tissue sections were deparaffinized in xylene and rehydrated in graded series of ethanols followed by heat induced epitope retrieval in citrate buffer (PH 6.0). Mcl-1 expression were detected using a primary antibody against Mcl-1(Abcam, Cambridge, MA; 1:200). After incubation with a biotinylaed secondary antibody and DAB (Dako, Carpenteria, CA), the slides were rinsed and counterstained with Mayer‘ hematoxylin.

### Statistical analysis

Two-sided Student’s *t* test was used to analyze the differences in miR-20a expression [17], proliferation, colony formation number, percent of cells in respective cell cycle and apoptotic rate. Data were presented as mean ± SD from at least three separate experiments. The Fisher exact test was used for analysis of categorical data. Association of miR-20a expression with overall survival (OS) and recurrence-free survival (RFS) was estimated by Kaplan-Meier method, and the resulting curves were compared using the log-rank test. The multivariate Cox proportional hazard regression analysis were used to evaluate the contribution of independent prognostic factors to patient’s survival by only taking the factors as covariates, that were found to be significant in univariate analysis.

Overall survival was calculated as the interval between the date of the LT and either the date of death or the last follow-up date of the patient. Recurrence-free survival was calculated as the time from the date of LT until the date of tumor recurrence and was censored at the time of last following-up or death if at that time there was no evidence of tumor recurrence.

All statistical analyses were conducted using the SPSS version 17.0 (SPSS Inc. Chicago, IL). p <0.05 was considered statistically significant.

## Results

### MiR-20a was down-regulated in primary HCC tissues especially in those with tumor recurrence following LT

**Figure 1:**
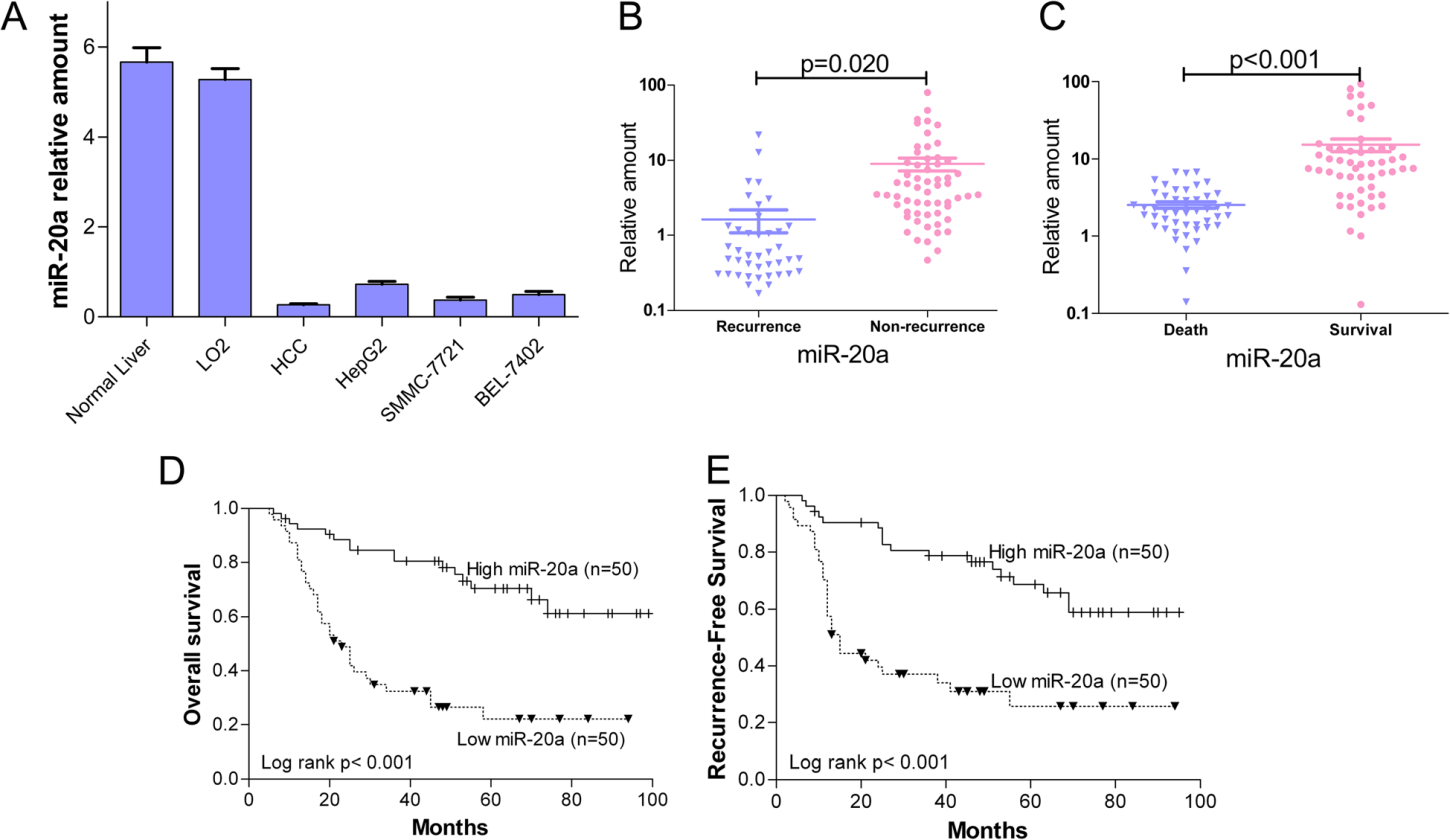
**Decrease expression of miR-20a in HCC is associated with tumor recurrence and poor prognosis following LT.** (**A**) Expression of miR-20a was measured in 100 FFPE HCC samples, 10 normal liver tissue, normal liver cell line LO2 and 3 HCC cell lines by qRT-PCR, and the expression levels of miR-20a were normalized to U6 RNA expression for subsequent analyses. (**B**) The expression levels of miR-20a were further down-regulated in HCC samples of patients with tumor recurrence after LT. (**C**) The expression levels of miR-20a were much lower in the patients who had died after LT than the patients who still survived. (**D** and **E**) Kaplan-Meier analyses of overall survival and recurrence-free survival in 100 patients with HCC following LT according to the expression levels of miR-20a.

With the purpose of revealing the expression and significance of miR-20a in HCC, we first detected the expression of miR-20a in 100 cases of HCC and 10 normal liver tissue by Taqman qPCR. The expression of miR-20a was significantly down-regulated in HCC tissue compared with normal liver tissue (*P* = 0.001; Figure 1A) and the expression levels of miR-20a were further down-regulated in HCCs samples of patients with tumor recurrence after LT (*P* = 0.020; Figure 1B). In accordance with the data between recurrence and non-recurrence patients, the expression of miR-20a was much lower in the patients who had died after LT than the patients who still survived (*P* < 0.001; Figure 1C). At the same time, we also detected the expression level of miR-20a in normal liver cell line, LO2, and three HCC cell lines, HepG2, SMMC-7721 and BEL-7402. We found that the expression level of miR-20a in HCC cell lines was lower than in LO2 cells, which was similar with the results of clinical HCC samples (Figure 1A).

### Decrease expression of miR-20a correlates with aggressive tumor features

The relationships between miR-20a expression and clinicopathological features were analyzed based on the miR-20a real-time PCR readings. As shown in Table 1, decrease expression of miR-20a in HCC was associated significantly with aggressive pathologic features, such as the largest tumor size (*P* = 0.014), multinodular HCC (*P* = 0.034) and micro-vascular invasion (*P* = 0.016).

### Decrease expression of miR-20a in HCC is associated with tumor recurrence and poor prognosis

To further explore the clinical relevance of miR-20a, Kaplan-Meier and univariate Cox proportional hazard regression analyses were performed. Kaplan-Meier analysis showed decrease miR-20a expression correlated with shorter overall survival (*P* < 0.001; Figure 1D) and recurrence-free survival (*P* < 0.001; Figure 1E) of HCC patients following LT. Similarly, univariate analysis showed that miR-20a expression was associated with OS (*P* = 0.009; Table 2) and RFS (*P* = 0.015; Table 3). The other significant prognostic factors associated with OS and RFS in univariate analyses were also shown in Tables 2 and 3.

**Table 2:** Univariate and multivariate Cox regression analyses of overall survival in 100 HCC patients following LT

**Parameter**	**Univariate analysis**			**Multivariable analysis**		
	**HR**	**95% CI**	***P*-value**	**HR**	**95% CI**	***P*-value**
Age	0.875	0.912-1.172	0.169	-	-	-
Gender	1.034	0.561-1.907	0.915	-	-	-
HBV infection	0.342	0.261-0.745	0.230	-	-	-
Cirrhosis	0.833	0.495-1.438	0.467	-	-	-
Tumor size	1.319	1.012-1.894	0.021*	1.175	0.981-1.857	0.035*
Tumor stage (III)	2.938	1.359-5.493	0.018*	2. 354	0.846-2.943	0.851
Histologic grade (G3/G1-2)	3.342	1.837-6.421	0.009*	1.773	0.732-3.101	0.082
Milan criteria (out)	1.756	1.043-3.433	0.017*	1.365	0.935-2.778	0.347
AFP >400 (ng/ml)	2.027	1.386-3.543	0.023*	1.569	1.031-4.603	0.031*
Micro-vascular invasion	3.739	1.929-6.758	0.005*	2.671	1.756-5.545	0.009*
miR-20a (low)	4.483	2.769-9.572	0.009*	4.937	2.221-9.503	0.022*

Note: *statistically significant difference.

**Table 3:** Univariate and multivariate Cox regression analyses of recurrence-free survival in 100 HCC patients following LT

**Parameter**	**Univariate analysis**			**Multivariable analysis**		
	**HR**	**95% CI**	***P*-value**	**HR**	**95% CI**	***P*-value**
Age	0.849	0.713-1.275	0.746	-	-	-
Gender	1.092	0.534-2.801	0.331	-	-	-
HBV infection	0.583	0.228-1.144	0.192	-	-	-
Cirrhosis	0.746	0.434-1.204	0.493	-	-	-
Tumor size	1.632	1.031-1.918	0.011*	1.253	1.123-1.792	0.014*
Tumor stage (III)	1.876	1.319-2.592	0.026*	1.348	0.935-1.813	0.365
Histologic grade (G3/G1-2)	3.731	1.774-5.103	0.024*	2.931	1.526-3.858	0.079
Milan criteria (out)	2.182	1.962-6.212	0.018*	1.935	1.332-3.563	0.156
AFP >400 (ng/ml)	1.939	1.638-4.809	0.012*	2.235	1.771-4.595	0.028*
Micro-vascular invasion	4.017	3.137-7.583	0.009*	3.643	2.964-6.927	0.012*
miR-20a (low)	4.591	2.933-8.457	0.015*	4.281	3.316-6.741	0.013*

Note: *statistically significant difference.

### MiR-20a independently predicts the survival of HCC patient following LT

To get insight into the survival prediction potential of miR-20a, we performed multivariate Cox proportional hazard regression analyses to test whether miR-20a expression was an independent prognostic factor associated with survival. Taking tumor size, tumor stage, histologic grade, Milan criteria, pre-LT serum AFP level, micro-vascular invasion and miR-20a as covariates, that were found to be significant in univariate analysis, we found that decrease miR-20a expression (HR = 4.937, *P* = 0.022; Table 2), tumor size (HR = 1.175, *P* = 0.035; Table 2), pre-LT serum AFP level (HR = 1.569, *P* = 0.031; Table 2) and micro-vascular invasion (HR = 2.671, *P* = 0.009; Table 2) were significantly associated with OS and that the prognostic value of miR-20a was independent the microvasculuar invasion.

Similarly, decrease miR-20a expression (HR = 4.281, *P* = 0.013; Table 3), tumor size (HR = 1.253, *P* = 0.014; Table 3), pre-LT serum AFP level (HR = 2.235, *P* = 0.028; Table 3) and micro-vascular invasion (HR = 3.643, *P* = 0.012; Table 3) significantly affected RFS of HCC patients following LT.

### Effects of miR-20a restoration on HCC cell proliferation and cell cycles in vitro

**Figure 2:**
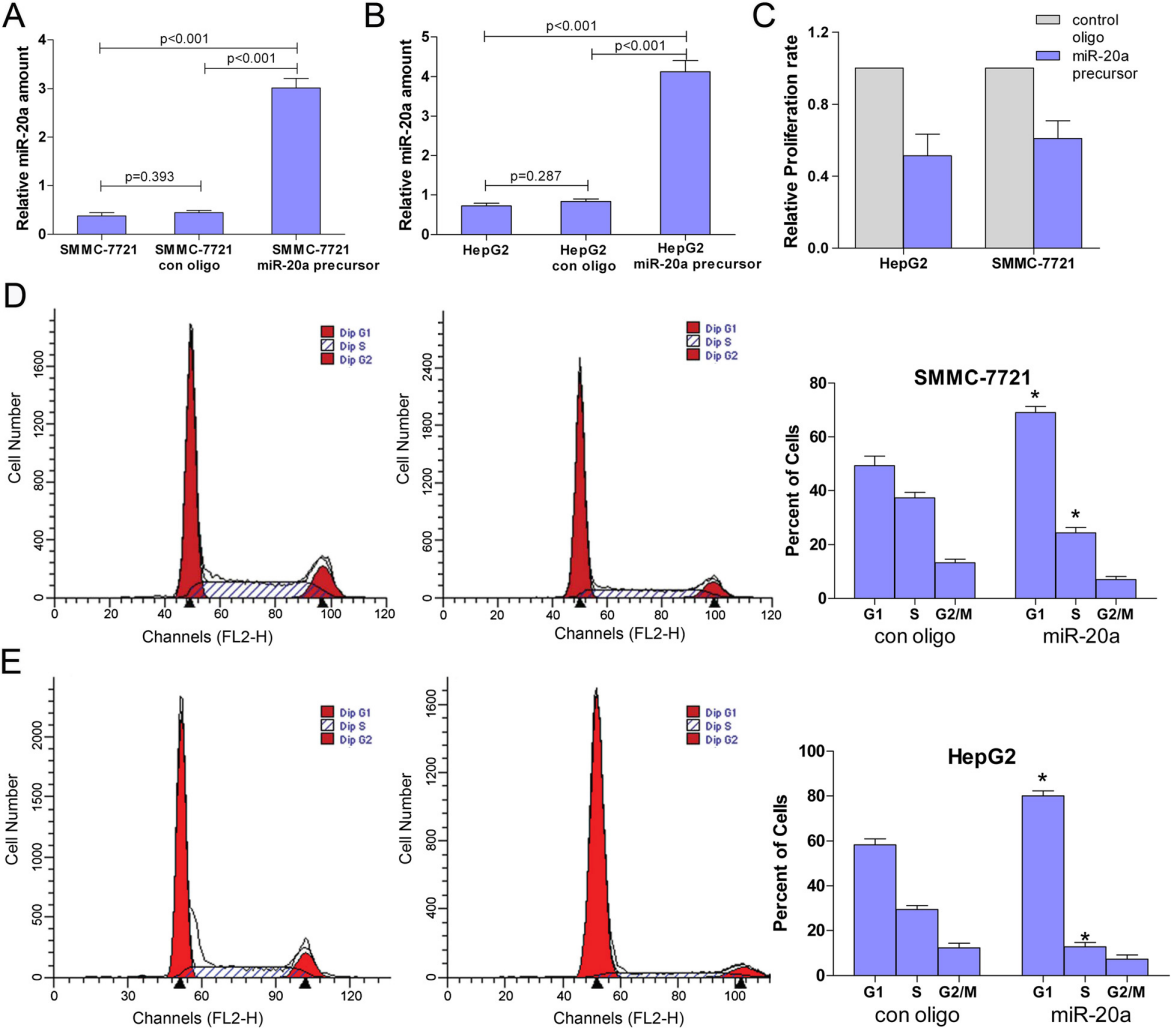
**MiR-20a restoration in HCC cell lines inhibit proliferation and block cell cycle progression in vitro (A) and (B) Validation of miR-20a level in SMMC-7721 and HepG2 cells upon transfection with miR-20a precursor.** (**C**) Proliferation assay of HCC cell lines in response to miR-20 restoration. HepG2 or SMMC-7721 cells were seeded into 96-well plates and incubated in the presence of miR-20a precursor or control oligonucleotide. Cell proliferation assay was done after culturing for 72 h. The experiment was done in triplicate. (**D**) and (**E**) Influence of miR-20a on cell cycle progression of HCC cell lines. SMMC-7721 and HepG2 cells were transfected with miR-20a precursor. Cell cycle analysis was performed by flow cytometry. Data are given as mean ± SD of three independent experiments. Asterisks indicate statistical significance of differences in the number of cells in G1, S, or G2/M between miR-20a precursor transfected and control oligo transfected cells (*P* < 0.05; Student’s *t* test).

Cell proliferation is a key determinant of tumor malignancy. However, the association of miR-20a with HCC cell proliferation is unknown. To investigate whether miR-20a up-regulation plays an important role in HCC cell proliferation, HepG2 and SMMC-7721 cells were transfected with miR-20a precursor and the effects of miR-20 restoration were detected by Taqman qPCR prior to the proliferation assay (Figure 2A and B). In cell proliferation assay, the proliferation rate was suppressed in HepG2 and SMMC-7721 cells after transfection with miR-20a precursor, and the inhibitory efficiencies were 41.3% and 39.0%, respectively (Figure 2C).

Cell cycle analysis was performed to determine whether the effect of miR-20a on cell proliferation of HepG2 and SMMC-7721 HCC cell lines was due to cell cycle arrest. The result showed that when comparing to the control oligonucleotide, the percentages of cells at G1 phase were increased in both HCC cell lines (for HepG2, from 58.3% to 80.0%, *P* = 0.003; for SMMC-7721, from 49.3% to 69.1%, *P* = 0.009), while the percentages of cells at S phase were decreased in HepG2 (from 29.3% to12.7%, *P* = 0.003) and SMMC-7721 (from 37.3% to 24.3%, *P* = 0.011) (Figure 2D and E). All of these data demonstrated that overexpression of miR-20a could induce the HCC cell cycle G1 arrest and block cell cycle progression. Disappointingly, the percentage of cells at G2/M phase was of no statistic significance in HepG2 or SMMC-7721 cells transfected with miR-20a when compared with the control group, although the absolute value was decreased to a certain extent (Figure 2D and E).

### MiR-20a restoration induces HCC cells to apoptosis

**Figure 3:**
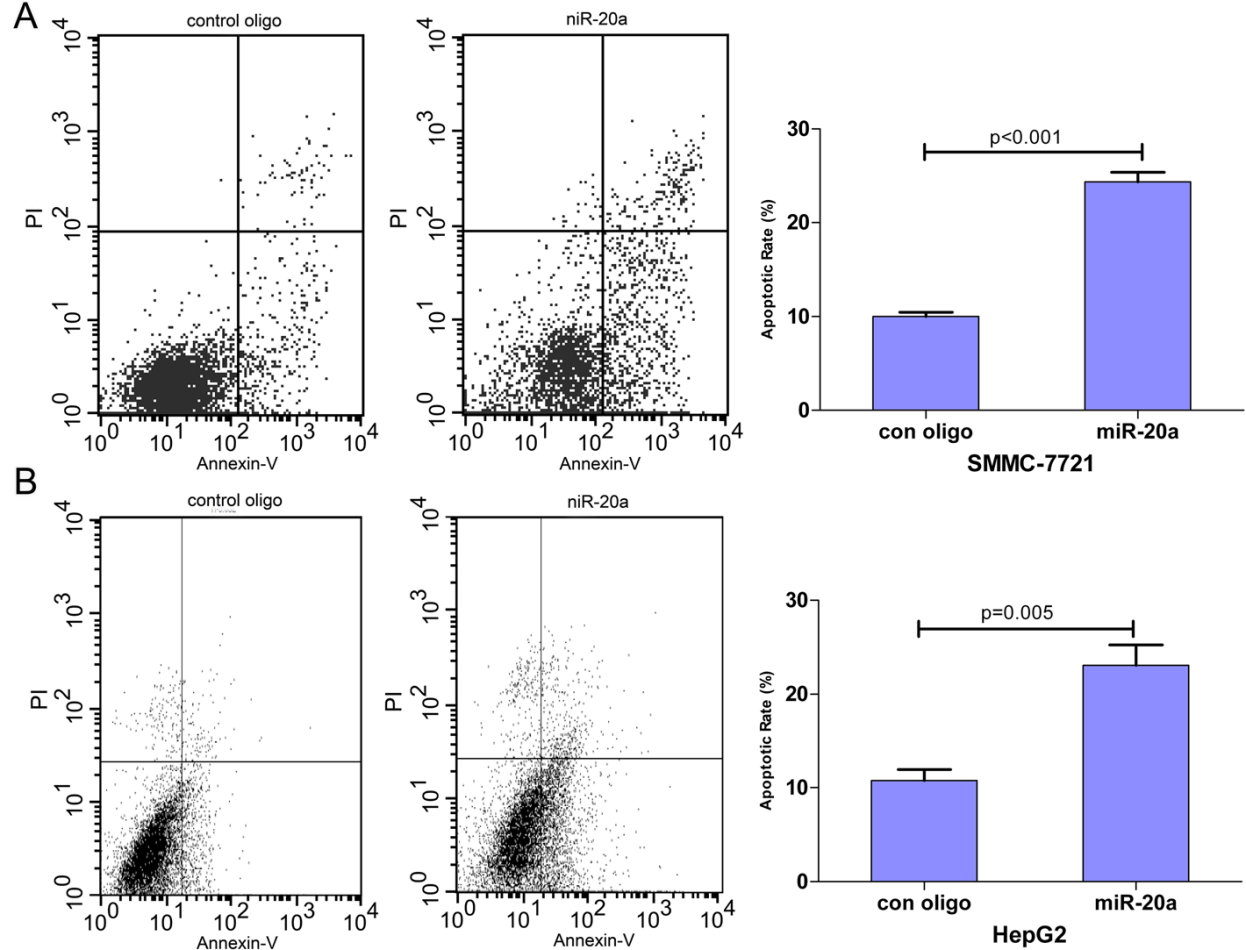
**MiR-20a restoration in HCC cell lines induces apoptosis a SMMC-7721 and HepG2 cells transfected with miR-20a precursor were stained with FITC and PI.** 20,000 cells were analyzed by flow cytometry. The LR quadrant represents the percentage of apoptotic cells (annexin V + and PI-) in the total cell population. Each type of cell was assayed in triplicate. All data were processed by Student’s *t* test and presented as mean ± SD. Asterisks indicate statistical significance of differences in the apoptosis rate of cells between miR-20a precursor transfected and control oligonucleotide transfected cells (*P* < 0.05; Student’s *t* test).

To better understand the effect of proliferation inhibition of miR-20a on HCC cells, we further investigated whether miR-20a could induce apoptosis of HCC cells. Flow cytometry analysis showed that much more apoptotic cells were observed in the miR-20a restoration group compared with the control group (Figure 3). Significant differences were observed both in SMMC-7721 (*P* < 0.001) and HepG2 (*P* = 0.005) HCC cells. The apoptosis rates increased from 10.1% to 24.1% for SMMC-7721 cells and from 12.9% to 23.1% for HepG2 cells after transfeted by miR-20 precursor.

### MiR-20a directly regulates Mcl-1 expresion

**Figure 4:**
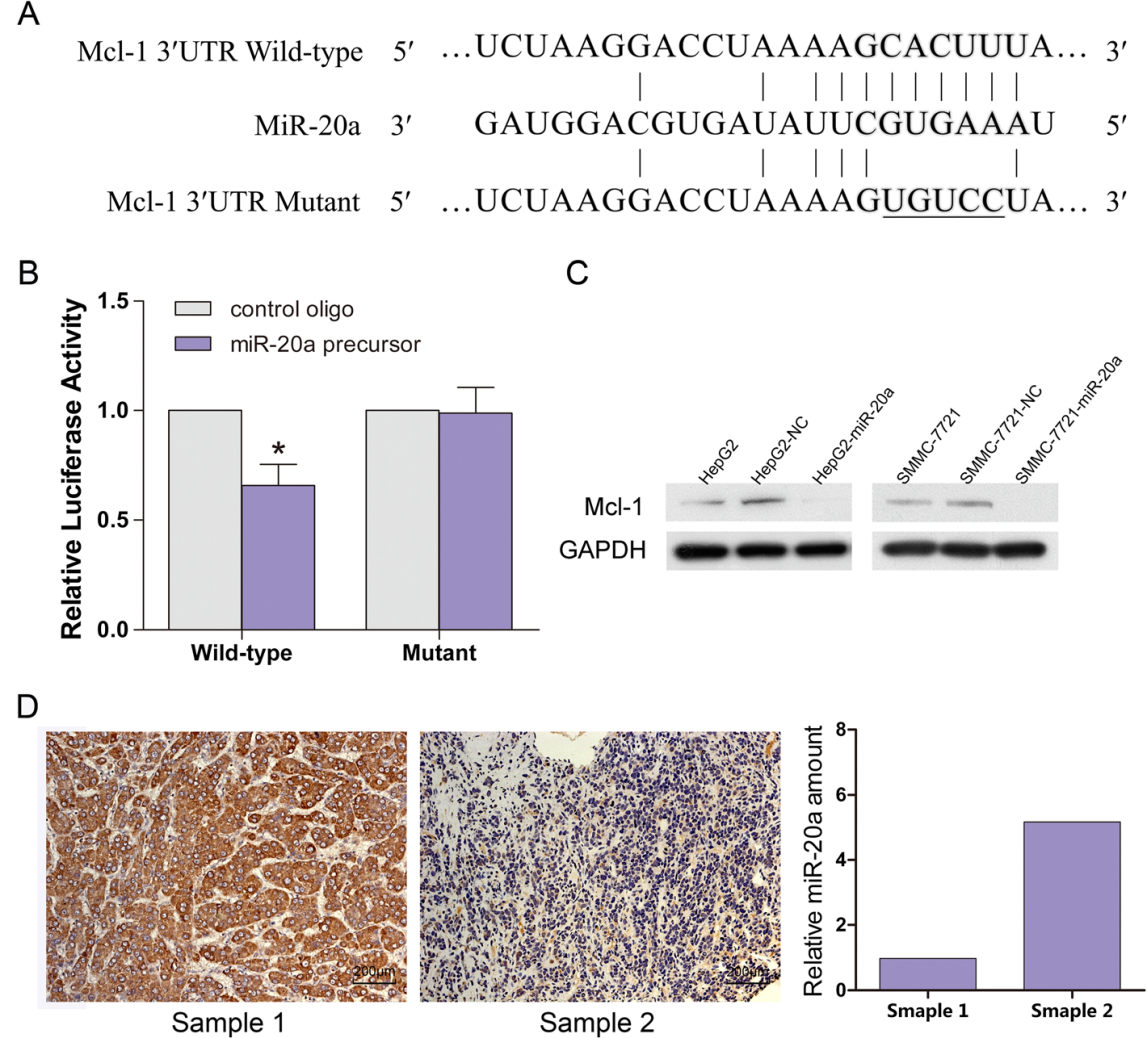
**MiR-20a directly regulates Mcl-1 expression.** (**A**) Wild-type and mutant of putative miR-20a target sequences of Mcl-1 3^′^UTR. (**B**) MicroRNA luciferase reporter assay. Wild type and mutant miR-20a target sequences were fused with luciferase reporter and cotransfected with miR-20a precusor or control oligo into HEK293T cells. The firefly luciferase activity of each sample was normalized to the Renilla luciferase activity. MiR-20a significantly suppressed the luciferase activity of wild-type Mcl-1 3^′^UTR (p = 0.027). (**C**) Effects of miR-20a overexpression on the level of cellular Mcl-1 in HepG2 and SMMC-7721 HCC cells without transfection or cells transfected with NC or miR-20a were analyzed by western blot. (**D**) Analysis of Mcl-1 and miR-20a expression in the same HCC tissue by IHC. Brown signal in IHC was considered as positive staining for Mcl-1. Scale bar = 200 μm.

The preceding findings indicated that miR-20a acted as a proliferation suppressor in HCC. Therefore, we then aimed to investigate the potential gene targets of miR-20a that contributed to its antiproferation functions. Potential target genes of miR-20a were first predicted using online databases (TargetScan, PicTar, and miRanda). Among them, Mcl-1 was chosen for further experimental validation, not only because it was identified as a target of miR-20a by all these three databases, but also due to its frequent overexpression in tumor tissues and well-known importance in the regulation of cell-cycle progression [18] and anti-apoptotic activity [19]. Dual-luciferase reporter analysis showed that coexpression of miR-20a significantly inhibited the activity of firefly luciferase that carried wildtype but not mutant 3^′^UTR of Mcl-1 (Figure 4A and B), indicating that miR-20a may suppress gene expression throuth its binding site at 3^′^UTR of Mcl-1. Moreover, introduction of miR-20a diminished the expression of cellular Mcl-1 protein expression in HepG2 and SMMC-7721 cells (Figure 4C). Consistently, HCC tissues with low miR-20a showed much higher Mcl-1 expression, compared with those with high miR-20a expression by IHC detection (Figure 4D). These findings indicated that miR-20a might negatively regulate the expression of Mcl-1 by directly targeting its 3^′^UTR.

## Discussion

Recently, attentions have focused on the role of microRNA regulation in essential mechanisms for cancer progression and metastasis, including proliferation, invasion, migration, angiogenesis and apoptosis. In human cancers, previous studies have also shown that dysregulation of certain microRNAs are associated with clinical outcomes of pancreatic cancer [20], breast cancer [21], lung adenocarcinoma [22], gastric cancer [23], and HCC [24]. A few reports even demonstrated that the expression profiling of microRNAs may be a more accurate method of classifying cancer subtype than using the expression profiles of protein-coding genes [6,25].

In the present study, we confirmed that the expression level of miR-20a was decreased in HCC tissues and three HCC cell lines. Loss expression of miR-20a was associated with poor survival and tumor recurrence in HCC patients who underwent LT. MiR-20a restoration could suppress cell proliferation by inhibiting cell cycle progression and inducing apoptosis in vitro. Moreover, we identified Mcl-1, which is an antiapoptotic member of Bcl-2 family, as a direct and functional target of miR-20a. To our best knowledge, this is the first study showing that miR-20a regulates cellular proliferation in HCC cell and correlates miR-20a to prognosis of HCC.

Although the expression of miR-20a is often down-regulated in HCC, it is significantly up-regulated in lung cancer [26], gliomas [9], and colon cancer [8]. This discrepancy is likely due to the target genes of miR-20a are different in different cancer cells and suggests that altered expression of this microRNA may have diverse effects in different tumor cells, either as an oncogene or a tumor suppressor.

Mcl-1 is an antiapoptotic member of Bcl-2 family and increased Mcl-1 protein level is commonly observed in various types of cancers, including HCC [27]. Depletion of Mcl-1 has been well proven to sensitize human HCC cancer cells to apoptosis [28]. Furthermore, overexpression of Mcl-1 is correlated with shorter survival of cancer patients [29]. All of these previous studies are consistent with our findings that decrease expression of miR-20a promotes HCC cell proliferation by targeting Mcl-1 which sensitizes HCC cells to apoptosis. According to many other published articles, Stat3, E2F family, cyclin-dependent kinase inhibitor CDKN1a/p21 and transforming growth factor-beta receptor 2 (TGFBR2) have also been identified as targets of miR-20a. In addition, miR-20a also targets transforming growth factor-beta receptor 2 (TGFBR2), which is a key mediator of TGF-β signaling and strongly implicated in human carcinogenesis [6]. Our identification of Mcl-1 as a target of miR-20a provides new insights into the mechanisms underlying HCC proliferation and resistance to apoptosis.

## Conclusions

We have shown that miR-20a was decreased in HCC tissues and the expression level of miR-20a is a significant prognostic factor for HCC patients. MiR-20a restoration inhibited HCC cell proliferation and induced apoptosis by directly targeting Mcl-1 3^′^UTR. Our data not only supply novel insights regarding miR-20a function and the potential mechanisms of HCC cell proliferation, but also suggest miR-20a may serve as a potential therapeutic target and biomarker for survival of HCC patients following LT.

## Abbreviations

miR-20a: microRNA-20a; HCC: Hepatocellular carcinoma; LT: Liver transplantation; Mcl-1: Myeloid cell leukemia sequence 1; 3′-UTR: 3^′^-untranslated region; FFPE: Formalin-fixed paraffin-embedded; AFP: Alpha-fetoprotein; PCR: Polymerase chain reaction; PI: Propidium idide; annexin V–FITC: Annexin V–fluoresein isothiocyanate; IHC: Immunohistochemical; OS: Overall survival; RFS: Recurrence-free survival.

## Competing interests

The authors declare that they have no competing interests.

## Authors’ contributions

MQF and CBH participated in the study design, conducted the real-time PCR assays and drafted the manuscript; YG carried out the proliferation and flow cytometry analysis; YX carried out the luciferase reporter and western bolt assay; JXS conducted immunohistochemical staining; LZ conceived of the study, and participated in its design and coordination, and reviewed the manuscript. All authors read and approved the final manuscript.
